# Enuresis

**Published:** 1905-04-29

**Authors:** 


### ENURESIS.

The number of cures vaunted from time to time for this distressing condition speaks volumes for its occasional recalcitrancy. Dr. Percy Lewis [1] enunciates a conviction that most cases depend upon an excess of carbohydrates in the diet, and claims a large measure of success for a more or less strict

"diabetic" régime. Dr. J. R. Clemens[2] summarises the four chief causes of urinary incontinence in children as follows: (1) Hyperacidity of the urine (incontinence both nocturnal and diurnal); (2) adenoids (incontinence nocturnal only); (3) chronic interstitial nephritis (incontinence nocturnal and diurnal); (4) alkalinity of urine (incontinence nocturnal and diurnal).

Few physicians of experience, we fancy, will accept this facile docketing, not to mention the statement that chronic interstitial nephritis is common in childhood, a dictum opposed to all clinical and pathological experience in this country at all events. The first, second and fourth items are generally recognised as possible exciting causes of the malady, but they are far from exhausting the list. "There are few conditions," says Dr. Goodhart, "which require more careful investigation than this, and few in which such a variety of circumstances may conspire to bring it about. Granting that it is due to a nervous fault, the results of treatment would seem to show that sometimes it is due to hypersensitiveness of the centre, sometimes to deficiency of the natural delicacy of perception either on the part of the lumbar cord or the higher centres to which it should transmit its own knowledge. In some cases the constitutional build of the patient must be considered: the sleeping habits of the nervous system, the question of developing sexual sensation, the condition of prepuce, urethra, rectum; the possibility of the existence of local disease, the presence of ascarides, the condition of the urine, and, in confirmed cases, the question of habit."

This judicial statement represents the concensus of the best opinion. Resolved into terms of practice, it is an indictment of rule-of-thumb medication, which when unsuccessful, as is too frequently the case, merely gives the malady time to become inveterate by calling in the assistance of habit. It further points the way for a rational attack upon the condition, firstly by the removal of sources of local irritation if they exist, and secondly in their absence, by the fortifying of the nervous mechanism. Thus intestinal parasites must be looked for, and dislodged when found; circumcision must be performed if the prepuce come under suspicion; the hyperacid urine must be neutralised by administration of bicarbonate of soda, or citrate of potash, and excessive alkalinity corrected by benzoic or salicylic acid; any existing cystitis must receive attention, and the rare possibility of diabetes mellitus or insipidus not forgotten. In addition the heavy sleeper should be given a hard bed, and no more covering than is sufficient for bare comfort, fluid should be withheld towards evening, and the general vigour increased by hot-bathing followed by tepid douching and brisk friction. Lastly comes the question of drugs. Belladonna will cure many cases, but may require to be pushed to the verge of excess before it becomes effective. A trial of large doses should always precede the rejection of this drug as useless. Other cases yield to bromide of potash and chloral hydrate. Unna recommends liquid extract of rhus aromatica in doses of 10 minims thrice daily for a child of five years or thereabouts. Dr. Goodhart speaks well of ergot, given in doses of 20 minims of the liquid extract for a child of similar age. Dr. Coutts advocates the tincture of lycopodium in doses of 20 minims to half a drachm; and Dr. Eustace Smith is in the habit of adding to belladonna and the tincture of lycopodium doses of tincture of cantharides (one minim) and salicylate of soda. Finally, it has been advised that where the bladder, from long habit, is reduced in capacity, dilation of this viscus with water should be practised under an anæsthetic.

[1] Brit. Journ. of Children's Diseases, Feb. 1905. [2] Archives of Pediatrics. New York, March 1905.

